# Skin bacterial microbiome of a generalist Puerto Rican frog varies along elevation and land use gradients

**DOI:** 10.7717/peerj.3688

**Published:** 2017-08-29

**Authors:** Myra C. Hughey, Janelle A. Pena, Roberto Reyes, Daniel Medina, Lisa K. Belden, Patricia A. Burrowes

**Affiliations:** 1 Department of Biological Sciences, Virginia Polytechnic Institute and State University (Virginia Tech), Blacksburg, VA, United States of America; 2 Department of Biology, Universidad de Puerto Rico, San Juan, Puerto Rico

**Keywords:** Elevation, Land use, *Batrachochytrium dendrobatidis*, Chytrid, Skin bacteria, Microbiome, *Eleutherodactylus coqui*

## Abstract

Host-associated microbial communities are ubiquitous among animals, and serve important functions. For example, the bacterial skin microbiome of amphibians can play a role in preventing or reducing infection by the amphibian chytrid fungus, *Batrachochytrium dendrobatidis*. Evidence suggests that environmental bacteria likely serve as a source pool for at least some of the members of the amphibian skin bacterial community, underscoring the potential for local environmental changes to disrupt microbial community source pools that could be critical to the health of host organisms. However, few studies have assessed variation in the amphibian skin microbiome along clear environmental gradients, and so we know relatively little about how local environmental conditions influence microbiome diversity. We sampled the skin bacterial communities of Coqui frogs, *Eleutherodactylus coqui* (*N* = 77), along an elevational gradient in eastern Puerto Rico (0–875 m), with transects in two land use types: intact forest (*N* = 4 sites) and disturbed (*N* = 3 sites) forest. We found that alpha diversity (as assessed by Shannon, Simpson, and Phylogenetic Diversity indices) varied across sites, but this variation was not correlated with elevation or land use. Beta diversity (community structure), on the other hand, varied with site, elevation and land use, primarily due to changes in the relative abundance of certain bacterial OTUs (∼species) within these communities. Importantly, although microbiome diversity varied, *E. coqui* maintained a common core microbiota across all sites. Thus, our findings suggest that environmental conditions can influence the composition of the skin microbiome of terrestrial amphibians, but that some aspects of the microbiome remain consistent despite environmental variation.

## Introduction

All animals host a diverse community of microbes. These microbes, along with their genetic contributions, are termed the microbiome, and they provide many important processes for their hosts (McFall-Ngai et al., 2013). For example, the vertebrate gut microbiome helps regulate development of the immune system, as well as playing critical roles in digestion and preventing pathogen colonization (Shreiner, Kao & Young, 2015). Transmission of these microbial symbionts can occur in several ways. In some systems, highly specialized symbionts are vertically transmitted from mother to offspring (Funkhouser & Bordenstein, 2013), while in other systems, many symbionts appear to be obtained and maintained from environmental sources (Bright & Bulgheresi, 2010). For species that obtain symbionts from environmental source pools, variation in environmental conditions that impact those source pools could alter the composition, and ultimately the function, of these microbial communities.

Large-scale differences in environmental conditions contribute to variation in local microbial communities (Fierer & Jackson, 2006), including those that might colonize hosts. For instance, conversion from natural to agricultural land use alters a variety of soil properties, such as temperature and moisture, which in turn impacts the diversity and composition of microorganisms present in soil (Matson et al., 1997). In addition, elevational gradients in soil pH appear to play role in determining the composition of soil bacterial assemblages on mountains (Shen et al., 2013). Despite the known impact of abiotic factors on microbial source pools, relatively little work has focused on understanding variation in the microbiome of wildlife across environmental gradients. Studies of non-human primates and frogs have found changes in the gut microbiome associated with different land use types (e.g., natural versus human-modified habitats), seemingly due to differences in the diet of animals in those habitats (Amato et al., 2013; Chang et al., 2016). In both systems, the gut microbiomes of hosts inhabiting the anthropogenically-modified habitats had characteristics that could negatively impact host health (Amato et al., 2013; Barelli et al., 2015; Gomez et al., 2015; Chang et al., 2016).

The skin microbiome of amphibians is receiving substantial research attention, in part because of its potential role as a barrier against pathogen infection (Woodhams et al., 2016). Variation in amphibian skin microbiome diversity among individuals, populations, and species is well-documented (McKenzie et al., 2012; Kueneman et al., 2014; Walke et al., 2014; Belden et al., 2015; Rebollar et al., 2016). However, the underlying causes and potential consequences of this variation are only just beginning to be understood. Importantly, large-scale factors, such as land use (Krynak, Burke & Benard, 2016) and elevation (Bresciano et al., 2015; Muletz-Wolz et al., 2017), can play a role in determining amphibian skin microbiome diversity. This is not altogether surprising, given that at least a portion of the amphibian skin microbiome is likely obtained from their surroundings and that environmental source pools of microbes appear to be critical for the maintenance of these skin communities (Fitzpatrick & Allison, 2014; Loudon et al., 2014; Michaels, Antwis & Preziosi, 2014; Walke et al., 2014; Rebollar et al., 2016). In addition, skin microbial diversity along these environmental gradients may be influenced by interactions with the chytrid fungus, *Batrachochytrium dendrobatidis* (*Bd*; Longcore, Pessier & Nichols, 1999), a widespread amphibian skin pathogen. *Bd* infection prevalence and intensity vary with elevation (Burrowes, Longo & Rodríguez, 2008; Burrowes et al., 2008; Sapsford, Alford & Schwarzkopf, 2013) and land use (Becker & Zamudio, 2011), and several recent studies have demonstrated a relationship between the presence of *Bd* and skin bacterial diversity (Jani & Briggs, 2014; Bresciano et al., 2015; Longo et al., 2015; Rebollar et al., 2016; Longo & Zamudio, 2017).

Our aim in this study was to assess variation in the skin microbiome of Coqui frogs, *Eleutherodactylus coqui*, along land use and elevation gradients. Specifically, we investigated elevational changes in the bacterial communities of the skin along two land use gradients (intact forest transect vs. disturbed forest transect) on the northern slopes of the Sierra de Luquillo in Puerto Rico. *Eleutherodactylus coqui* is a well-studied, terrestrial, direct-developing frog native to Puerto Rico (Joglar, 1998; Longo & Burrowes, 2010). It is an excellent model species for this study because it is a habitat generalist that can be found in abundance across a range of habitat types and elevations. In addition, *E. coqui* is vulnerable to *Bd*, but has persisted in spite of marked declines in the 1990s at high elevation sites in Puerto Rico (Burrowes, Joglar & Green, 2004).

## Methods and Materials

### Sampling and site selection

All sampling took place within one week during the warm-wet season in Puerto Rico in June 2014, between 1,900 and 2,200 h. Sampling sites were chosen based on elevation and land use. Overall, seven sites were identified, four in the “intact” forest of El Yunque and three in the “disturbed” zone of the northern face of the Sierra de Luquillo. El Yunque National Forest has been preserved since 1876, and protected by the US Forest Service since 1905. It is characterized by an elevation continuum of dense Tabonuco (*Dacryodes excels*), Palo Colorado (*Cyrilla racemiflora*), and Palma de Sierra (*Prestoea montana*) trees that lead up to a fog-covered elfin forest. The northern slopes of Sierra de Luquillo outside the National Forest consist of secondary forest fragmented by human settlements and farming activities. The presence of exotic tropical plants and fruit trees is very common. Sample sites across intact and disturbed forest were matched for three elevations: low (<200 m), mid (300–500 m), and high (>600 m) (Table 1). We also sampled one site at an even higher elevation (875 m) within the National Forest; however, for this site there was no elevation match within the disturbed forest (Table 1).

**Table 1:** Sites sampled during a field survey assessing the diversity of bacterial communities on the skin of Coqui frogs (*Eleutherodactylus coqui*). Name, land use type, elevation zone, elevation, and location of sampled sites, sample sizes, and sampling dates are provided. For sample sizes, *N* refers to the total sample size, and F/G/M/S refers to nongravid females, gravid females, males, and subadults, respectively. Elevation zone refers to the category that localities were assigned to for analyses of elevation, which excluded Bosque Enano at the highest elevation because there was no matching site in the “disturbed” transect.

Site	Land use	Lat/Long	Elevation zone (m)	Elevation (m)	Sample size *N* (F/G/M/S)	Date sampled
Boca del Yunque	Intact	18°20.474′N 65°45.602′W	Low	162	10 (6/0/4/0)	6∕04∕2014
La Quebrada	Disturbed	18°20.153′N 65°51.400′W	Low	140	10 (2/6/2/0)	6∕02∕2014
La Coca	Intact	18°19.090′N 65°46.271′W	Mid	460	11 (2/1/8/0)	6∕03∕2014
Carretera 956	Disturbed	18°18.641′N 65°51.105′W	Mid	416	10 (7/2/1/0)	6∕02∕2014
Palo Colorado	Intact	18°17.993′N 65°47.104′W	High	657	11 (3/0/7/1)	6∕03∕2014
Pico del Toro	Disturbed	18°16.870′N 65°51.491′W	High	642	10 (1/2/5/2)	6∕02∕2014
Bosque Enano	Intact	18°17.863′N 65°47.662′W	-NA-	875	10 (5/0/5/0)	6∕03∕2014

### Sample collection

At each of the seven sites, 11 individual *E. coqui* were sampled (total *N* = 77). Clean gloves were worn during collection and swabbing of each frog. At the time of collection, frogs at a site were placed individually in sterile whirlpak bags until swabbing. The maximum time frame between collection and swabbing of any given frog was 2 h. We recorded mass, SVL and sex of all individuals. For skin bacterial and *Bd* sampling, each individual was rinsed in 50 ml of sterile DI water to remove any transient bacteria, and then swabbed with a sterile rayon swab (MW113; Medical Wire & Equipment, Corsham. Wiltshire, UK). Swabbing for each frog consisted of ten strokes along the ventral side, five strokes along each thigh and hind foot and one stroke on each hand. After sample collection in the field, swabs were placed inside sterile 1.5 ml microcentrifuge tubes on ice. Once back in the laboratory, they were stored at −80°C until further analysis.

### DNA extraction, amplification, and 16S rRNA gene amplicon sequencing

DNA was extracted from swabs using the DNeasy Blood & Tissue Kit (Qiagen, Inc., Valencia, CA, USA). We followed the manufacturer’s Quick-Start protocol, except that for Step 1, we added 180 µl lysis buffer solution (20 mg lysozyme/1 ml lysis buffer) to each tube and incubated at 37°C for 1 hr, and for Step 2, we added 25 μl proteinase K to each reaction in addition to 200 µl buffer AL, and incubated at 70°C for 30 min.

To characterize the taxonomic diversity of the microbial community, we amplified the V4 region of the 16S rRNA gene following Caporaso et al. (2012). Each 25 µl reaction contained: 11.5 µl PCR water, 10 µl 5Prime Hot Master Mix, 0.5 µl 515f forward primer, 0.5 µl 806r reverse primer which included an individual 12-base barcode sequence unique to each sample, and 2.5 µl genomic DNA. Thermocycler conditions were set as follows: a denaturation step of 94°C for 3 min, followed by 34 cycles at 94°C for 45 s, 50°C for 60 s, and 72°C for 90 s and a final extension step at 72°C for 10 min. PCRs were run in triplicate for each sample and the three triplicates were combined after amplification.

Amplified DNA was quantitated using a Qubit® 2.0 Flourometer with a dsDNA HS assay kit according the manufacturer’s guidelines (Life Technologies, Carlsbad, CA, USA). Samples were pooled by combining approximately 180 ng of each amplicon into a single tube, and then this pooled sample was cleaned using the QIAquick PCR purification kit according to the manufacturer’s instructions (Qiagen, Inc., Valencia, CA, USA). The pooled sample (final elution volume = 50 µl) was sent to the Molecular Biology Core Facilities of the Dana Farber Cancer Institute at Harvard University (Cambridge, MA, USA) for 16S rRNA gene amplicon sequencing on an Illumina MiSeq instrument using a 250 bp paired-end strategy with 10% PhiX added to account for low base diversity.

### Detection and quantification of *Bd*

*Bd* infection status was determined using the Taqman real-time PCR assay developed by Boyle et al. (2004). Specifically, we amplified the ITS1 and 5.8S region of the *Bd* genome using the species-specific primers ITS1-3 Chytr and 5.8S Chytr and the probe MGB2. DNA from the same extractions described above for the 16S rRNA gene amplicon sequencing was also used in these *Bd* assays. The *Bd* DNA standards were prepared by making serial dilutions for 1,000–0.1 zoospores genome equivalents of the Puerto Rican *Bd* strain JEL427. The samples were run in duplicate and considered positive when amplification above 0.1 was observed in both replicates.

### Bacterial community analysis

Sequences were processed using the Quantitative Insights Into Microbial Ecology pipeline (MacQIIME, v. 1.8.0; Caporaso et al., 2010a). Forward and reverse reads from the raw Illumina files were joined using Fastq-join (Aronesty, 2011). Demultiplexing and initial quality filtering were then completed, with default quality filters, except that no errors were allowed in the barcode, the maximum number of consecutive low quality base calls allowed was set at 10, and the minimum number of consecutive high quality base calls required to include a read as a fraction of total read length was set at 0.5.

Resulting filtered sequences were then imported into Geneious® (Biomatters, Ltd.), remaining PhiX was filtered out, and reads between 250 and 255 bp were exported. Sequences were assigned to de novo operational taxonomic units (OTUs, ∼bacterial species) based on 97% sequence similarity using the UCLUST method (Edgar, 2010). To represent each OTU, we used the most abundant sequence from each cluster. Representative sequences were aligned to the Greengenes 13_8 reference database (DeSantis et al., 2006) using PyNAST (Caporaso et al., 2010b). Taxonomy was assigned using the RDP classifier (Wang et al., 2007). A phylogenetic tree was constructed using FastTree (Price, Dehal & Arkin, 2009).

Prior to statistical analyses, we removed all Archaea, chloroplast, and mitochondrial sequences and removed all OTUs with fewer than 0.01% of the total number of reads (Bokulich et al., 2013). To minimize effects of variable sequencing depth, OTU relative abundances in the dataset were rarefied to a sequencing depth of 19,000 reads. As a result of rarefaction, 5 samples (1 frog from each of five different sites) with read counts below 19,000 were eliminated from the dataset. This resulted in a final dataset of 72 samples, with a total of 1,368,000 reads clustered into 1,087 OTUs. Sequence data have been submitted to the NCBI database under accession number SRX2986909.

All statistical analyses were conducted in R v. 3.1.2 (R Core Team, 2014) using the vegan package (v. 2.3-2; Oksanen et al., 2015) unless specified otherwise. We calculated alpha diversity of bacterial communities as Simpson index and Shannon index (Haegeman et al., 2013) and Faith’s phylogenetic diversity (picante v. 1.6.2; Kembel et al., 2010). We assessed differences in alpha diversity among sites, elevation (paired across land use types for three elevation zones, excluding one unpaired site from the highest elevation zone), land use, sex (male or female, excluding three juveniles), and reproductive status (gravid and nongravid females from only those sites where there were both: Carretera 956, La Coca Falls, La Quebrada, and Pico del Toro; Table 1). We used generalized linear models for analyses of site. We used generalized linear mixed models for analyses of elevation, land use, sex, and reproductive status, including “Site” as a random effect to account for nestedness of individuals from the same site. All predictor variables were categorical. Shannon index and phylogenetic diversity data were normally distributed (Lilliefors (Kolmogorov–Smirnov) test for normality, all *P* > 0.05; package nortest v. 1.0-2; Gross & Ligges, 2015); thus, we fitted models using an underlying Gaussian distribution and identity function (package lme4 v. 1.1.5; Bates et al., 2015). Values for Simpson index range between zero and one; thus, we fitted models using an underlying Beta distribution and logit function (package glmmADMB v 0.8.0; Fournier et al., 2012; Skaug et al., 2014).

We assessed beta diversity of bacterial communities using Jaccard (presence/absence based), Bray–Curtis (relative abundance based) and weighted Unifrac (relative abundance and phylogenetic distance based) distances. Prior to computing Jaccard and Bray–Curtis distance matrices, rarefied sequence data were converted to relative abundance values for each sample by dividing the number of sequence reads for each OTU by the total number of reads in the sample. We compared dissimilarity of frogs from the same sites to frogs from different sites using beta regression, running separate analyses for each dissimilarity index. Because our sample size for intersite dissimilarity greatly outnumbered those for intrasite dissimilarity (inter: *N* = 2,221, intra: *N* = 335), we repeated the analysis 999 times using random subsets of 100 individuals from each group without replacement. We report the mean *P* value resulting from all 999 runs. We tested for variation in beta diversity of bacterial communities among sites, elevation, land use, sex, and reproductive status (as above) using permutational multivariate analysis of variance (PERMANOVA; function adonis; Anderson, 2001) based on 999 permutations. Patterns of beta diversity in bacterial communities were visualized using non-metric multidimensional scaling.

Following significant PERMANOVA results, we used a variable screening technique called the K–S measure (Loftus et al., 2015) to identify the OTUs driving the variation across elevation and land use (analysed separately). In brief, this technique compares the empirical (relative abundance) distributions of each OTU across predefined groups (e.g., elevation or land use), allowing one to identify OTUs with the greatest differences in relative abundance between groups. The K–S measure ranges from 0 to 1, where values closer to one imply a greater difference between the distributions than values closer to zero. K–S measures were calculated for each OTU, and the values were plotted in descending order. We used natural breaks in the steepness of the slope of K–S measures as a cutoff for OTUs to retain (*sensu* Loftus et al., 2015; Belden et al., 2015). The appropriateness of the cutoff was verified using non-metric multidimensional scaling to visualize the ability of the selected subset of OTUs to identify our groups relative to the full dataset.

Lastly, we determined a core microbiome for *E. coqui* based on our samples. We defined the core microbiome as OTUs present on ≥95% of individuals, as in Longo et al. (2015).

This research was approved by the Departamento de Recursos Naturales y Ambientales in Puerto Rico (2015-IC-014) and the University of Puerto Rico’s Institutional Animal Use and Care Committee (001002–05–27–2014).

## Results

In total, we identified 1,087 OTUs from the skin of *E. coqui* (*N* = 72 individuals), with a range of 143–818 of those present on individual frogs (mean ± SD = 470 ± 153 OTUs/frog). Most OTUs were from the phyla Proteobacteria (55%), Actinobacteria (17%), Bacteroidetes (13%), and Firmicutes (7%).

There was no indication that any of the individuals we sampled were infected with *Bd*. Additionally, there were no significant differences between males and females, or between gravid and non-gravid females, in alpha or beta diversity metrics (Tables 2 and 3). Therefore, we included all individuals in subsequent analyses focused on site, elevation, and land use effects.

**Table 2:** Results of generalized linear models indicating if OTU alpha diversity measures (Simpson index, Shannon index, Faith’s Phylogenetic diversity) were similar or different for five variables of interest for individuals sampled during a field survey assessing the diversity of bacterial communities on *Eleutherodactylus coqui* skin. The reported test statistics are Chi-Square.

*Sex (excludes juveniles)*			
Simpson	*χ*^2^ = 1.32	*df* = 1	*p* = 0.2506
Shannon	*χ*^2^ = 0.9832	*df* = 1	*p* = 0.3214
Phylogenetic diversity	*χ*^2^ = 0.085	*df* = 1	*p* = 0.7706
*Reproductive status (excludes males and juveniles)*			
Simpson	*χ*^2^ = 0.5714	*df* = 1	*p* = 0.4497
Shannon	*χ*^2^ = 2.277	*df* = 1	*p* = 0.1313
Phylogenetic diversity	*χ*^2^ = 2.4369	*df* = 1	*p* = 0.1185
*Site*			
Simpson	*χ*^2^ = 14.45	*df* = 6	*p* = 0.02499
Shannon	*F* = 3.7068	*df* = 6,65	*p* = 0.003135
Phylogenetic diversity	*F* = 5.8823	*df* = 6,65	*p* < 0.001
*Elevation (excludes Bosque Enano)*			
Simpson	*χ*^2^ = 2.86	*df* = 2	*p* = 0.2393
Shannon	*χ*^2^ = 2.113	*df* = 2	*p* = 0.3475
Phylogenetic diversity	*χ*^2^ = 2.467	*df* = 2	*p* = 0.2913
*Land use*			
Simpson	*χ*^2^ = 0.292	*df* = 1	*p* = 0.5889
Shannon	*χ*^2^ = 0.1168	*df* = 1	*p* = 0.7325
Phylogenetic diversity	*χ*^2^ = 0.2502	*df* = 1	*p* = 0.6169

**Table 3:** Results of permutational analysis of variance tests indicating if OTU beta diversity measures (Jaccard, Bray–Curtis, or weighted Unifrac) were similar or different for five variables of interest for individuals sampled during a field survey assessing the diversity of bacterial communities on *Eleutherodactylus coqui* skin. Pseudo-*F* test statistics and *R*^2^ values are reported.

*Sex (excludes juveniles)*				
Jaccard	*F* = 1.5872	*df* = 1,67	*p* = 0.127	*R*^2^ = 0.02314
Bray–Curtis	*F* = 1.466	*df* = 1,67	*p* = 0.273	*R*^2^ = 0.02141
Unifrac	*F* = 1.7335	*df* = 1,67	*p* = 0.094	*R*^2^ = 0.02522
*Reproductive status (excludes males and juveniles)*				
Jaccard	*F* = 0.90786	*df* = 1,21	*p* = 0.946	*R*^2^ = 0.04144
Bray–Curtis	*F* = 0.75772	*df* = 1,21	*p* = 0.874	*R*^2^ = 0.03483
Unifrac	*F* = 0.63926	*df* = 1,21	*p* = 0.82	*R*^2^ = 0.02954
*Site*				
Jaccard	*F* = 3.7965	*df* = 6,65	*p* = 0.001	*R*^2^ = 0.25951
Bray–Curtis	*F* = 3.1151	*df* = 6,65	*p* = 0.001	*R*^2^ = 0.22333
Unifrac	*F* = 2.3197	*df* = 6,65	*p* = 0.001	*R*^2^ = 0.17636
*Elevation (excludes Bosque Enano)*				
Jaccard	*F* = 3.9882	*df* = 2,59	*p* = 0.001	*R*^2^ = 0.11909
Bray–Curtis	*F* = 2.998	*df* = 2,59	*p* = 0.001	*R*^2^ = 0.09225
Unifrac	*F* = 2.3725	*df* = 2,59	*p* = 0.006	*R*^2^ = 0.07444
*Land use*				
Jaccard	*F* = 3.3976	*df* = 1,70	*p* = 0.001	*R*^2^ = 0.04629
Bray–Curtis	*F* = 3.222	*df* = 1,70	*p* = 0.003	*R*^2^ = 0.04401
Unifrac	*F* = 2.1472	*df* = 1,70	*p* = 0.03	*R*^2^ = 0.02976

**Figure 1:**
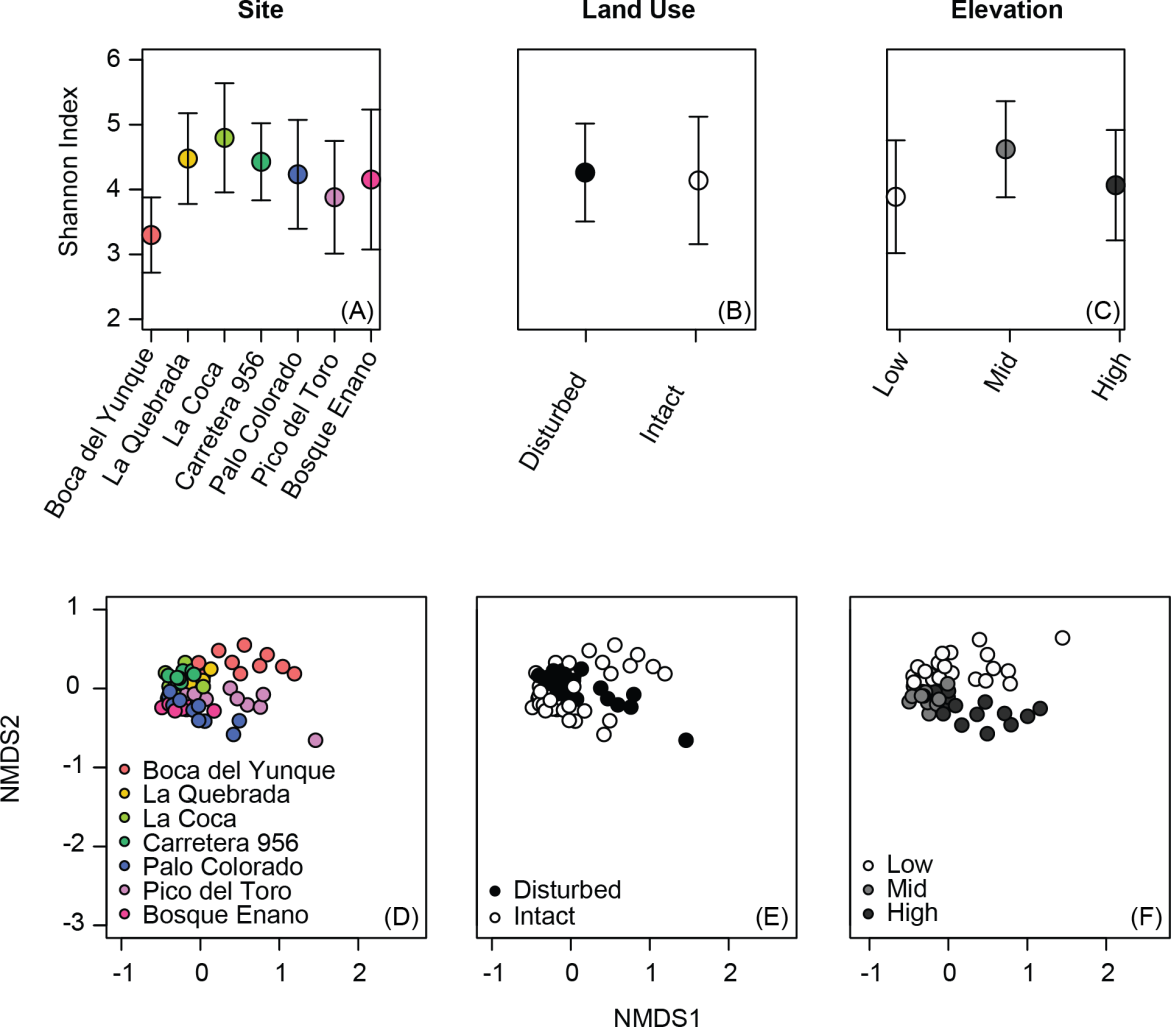
Variation in alpha diversity (A–C) and beta diversity (D–F) of the bacterial communities on the skin of Coqui frogs (*Eleutherodactylus coqui*) from seven sites varying in elevation and land use. Analyses of elevation (C, F) exclude one unpaired site from the highest elevation zone (Bosque Enano). Ordinations were constructed using non-metric multidimensional scaling based on Jaccard dissimilarities of relative abundance data (stress = 0.15 for D, E and also 0.15 for F).

Alpha diversity metrics varied among sites (Simpson index, Shannon index, phylogenetic diversity: all *P* < 0.02), but not based on elevation or land use (Table 2, Figs. 1A–1C). Although alpha diversity was variable at all sites, one site had particularly low alpha diversity relative to all other sites: Boca del Yunque (low elevation, intact forest; Fig. 1A).

Individuals displayed community structures ranging from relatively similar to extremely different both within and across sites. Indeed, the range for within site variation was comparable to that for between site variation (range, from the same site, Jaccard: 0.32–0.86, Bray–Curtis: 0.13–0.95, Unifrac: 0.10–0.97; from different sites, Jaccard: 0.33–0.90, Bray–Curtis: 0.18–0.99, Unifrac: 0.13–0.97). Still, the skin bacterial communities of individuals from the same site tended to be more similar in comparison to those from different ponds, at least in terms of OTU presence/absence and relative abundance (Jaccard and Bray–Curtis: *P* < 0.0001 and *P* = 0.03, respectively; Unifrac: *P* = 0.23).

**Table 4:** K–S measures and taxonomic information for 20 OTUs that best defined the differences in skin bacterial community structure across land use types (Intact and Disturbed Forest). The K–S measure ranges from 0 to 1, where values closer to one imply a greater difference between the K distributions than values closer to zero. OTUs are listed in order of descending K–S measure. Most OTUs were unclassified at the species level; if species information was available, it is listed along with the genus in the Genus column.

OTU	Phylum	Class	Order	Family	Genus	K–S measure
X575533	Actinobacteria	Acidimicrobiia	Acidimicrobiales	Unclassified	Unclassified	0.293
denovo67942	Bacteroidetes	Cytophagia	Cytophagales	Cytophagaceae	*Spirosoma*	0.286
X711526	Actinobacteria	Actinobacteria	Actinomycetales	Micrococcaceae	Unclassified	0.279
X879040	Proteobacteria	Alphaproteobacteria	Sphingomonadales	Sphingomonadaceae	Unclassified	0.274
X806726	Proteobacteria	Alphaproteobacteria	Rhizobiales	Methylocystaceae	Unclassified	0.271
X4302904	Bacteroidetes	Bacteroidia	Bacteroidales	Bacteroidaceae	*Bacteroides*	0.267
X4473756	Actinobacteria	Actinobacteria	Actinomycetales	Cellulomonadaceae	*Cellulomonas*	0.267
X4331180	Proteobacteria	Alphaproteobacteria	Sphingomonadales	Sphingomonadaceae	*Sphingomonas wittichii*	0.262
denovo72381	Actinobacteria	Actinobacteria	Actinomycetales	Unclassified	Unclassified	0.262
X239649	Acidobacteria	Acidobacteriia	Acidobacteriales	Acidobacteriaceae	Unclassified	0.257
X104265	Actinobacteria	Actinobacteria	Actinomycetales	Unclassified	Unclassified	0.257
X824146	Proteobacteria	Alphaproteobacteria	Sphingomonadales	Sphingomonadaceae	*Kaistobacter*	0.257
denovo37797	Proteobacteria	Betaproteobacteria	Burkholderiales	Alcaligenaceae	Unclassified	0.255
X154314	Proteobacteria	Alphaproteobacteria	Rhodospirillales	Acetobacteraceae	Unclassified	0.252
denovo12362	Proteobacteria	Alphaproteobacteria	Caulobacterales	Unclassified	Unclassified	0.252
X13226	Actinobacteria	Actinobacteria	Actinomycetales	Mycobacteriaceae	*Mycobacterium celatum*	0.248
X1097610	Proteobacteria	Alphaproteobacteria	Rhizobiales	Beijerinckiaceae	*Beijerinckia*	0.248
X742260	Acidobacteria	Acidobacteriia	Acidobacteriales	Acidobacteriaceae	*Edaphobacter*	0.248
X814864	Cyanobacteria	Nostocophycideae	Nostocales	Nostocaceae	Unclassified	0.248
X4399333	Proteobacteria	Betaproteobacteria	Unclassified	Unclassified	Unclassified	0.245

**Figure 2:**
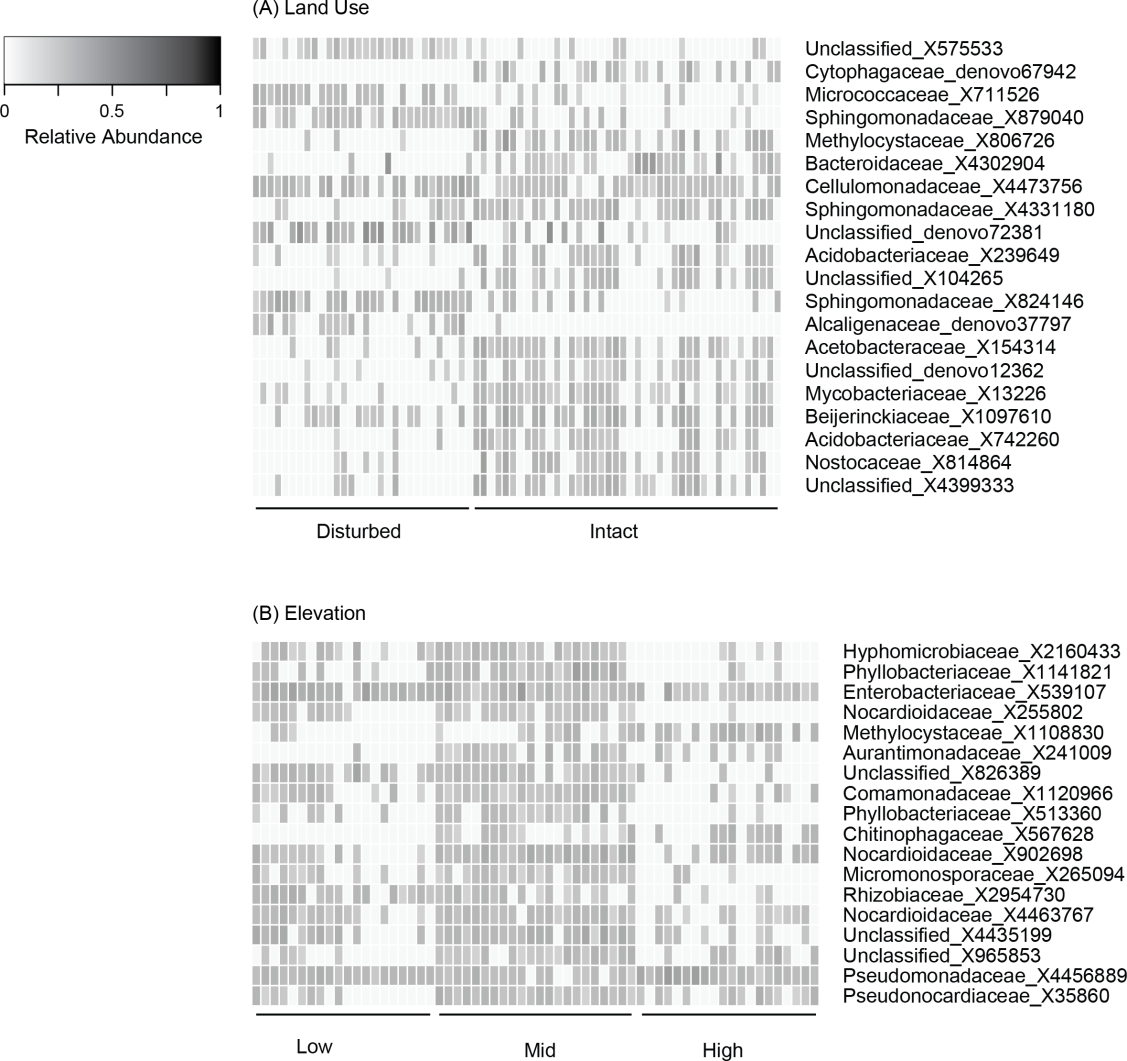
Relative abundance of OTUs selected based on K–S measures that best defined the differences in skin bacterial community structure of *Eleutherodactylus coqui* across (A) land use types and (B) elevations. OTU relative abundances ranged from 0 to 0.29. Lighter shades indicate lower relative abundances (white relative abundance = 0) and darker shades indicate higher relative abundances (darkest relative abundance = 0.29). OTUs are ordered top to bottom based on K–S measures (see Tables 4 and 5 for values and additional taxonomic information for each OTU).

**Table 5:** K–S measures and taxonomic information for 18 OTUs that best defined the differences in skin bacterial community structure among elevations (Low, Mid, and High). The K–S measure ranges from 0 to 1, where values closer to one imply a greater difference between the K distributions than values closer to zero. OTUs are listed in order of descending K–S measure. All OTUs were unclassified at the species level.

OTU	Phylum	Class	Order	Family	Genus	K–S measure
X2160433	Proteobacteria	Alphaproteobacteria	Rhizobiales	Hyphomicrobiaceae	*Devosia*	0.577
X1141821	Proteobacteria	Alphaproteobacteria	Rhizobiales	Phyllobacteriaceae	Unclassified	0.558
X539107	Proteobacteria	Gammaproteobacteria	Enterobacteriales	Enterobacteriaceae	*Pantoea*	0.534
X255802	Actinobacteria	Actinobacteria	Actinomycetales	Nocardioidaceae	*Nocardioides*	0.526
X1108830	Proteobacteria	Alphaproteobacteria	Rhizobiales	Methylocystaceae	Unclassified	0.506
X241009	Proteobacteria	Alphaproteobacteria	Rhizobiales	Aurantimonadaceae	Unclassified	0.506
X826389	Proteobacteria	Alphaproteobacteria	Rhizobiales	Unclassified	Unclassified	0.500
X1120966	Proteobacteria	Betaproteobacteria	Burkholderiales	Comamonadaceae	Unclassified	0.496
X513360	Proteobacteria	Alphaproteobacteria	Rhizobiales	Phyllobacteriaceae	*Mesorhizobium*	0.493
X567628	Bacteroidetes	[Saprospirae]	[Saprospirales]	Chitinophagaceae	Unclassified	0.491
X902698	Actinobacteria	Actinobacteria	Actinomycetales	Nocardioidaceae	Unclassified	0.488
X265094	Actinobacteria	Actinobacteria	Actinomycetales	Micromonosporaceae	Unclassified	0.484
X2954730	Proteobacteria	Alphaproteobacteria	Rhizobiales	Rhizobiaceae	*Agrobacterium*	0.480
X4463767	Actinobacteria	Actinobacteria	Actinomycetales	Nocardioidaceae	Unclassified	0.479
X4435199	Proteobacteria	Alphaproteobacteria	Sphingomonadales	Unclassified	Unclassified	0.478
X965853	Actinobacteria	Thermoleophilia	Solirubrobacterales	Unclassified	Unclassified	0.472
X4456889	Proteobacteria	Gammaproteobacteria	Pseudomonadales	Pseudomonadaceae	*Pseudomonas*	0.471
X35860	Actinobacteria	Actinobacteria	Actinomycetales	Pseudonocardiaceae	*Pseudonocardia*	0.468

**Table 6:** OTUs (*N* = 34) representing the core microbiota for *Eleutherodactylus coqui*. To be considered part of the core, OTUs had to be present on at least 95% of individuals. OTUs are grouped by the proportion of individuals they were present on: 100%, 99%, etc. For each OTU, taxonomic classification (Phylum, Class, Order, Family, Genus) and the mean ± SD relative abundance of each OTU across individuals are provided. Most OTUs were unclassified at the species level; if species information was available, it is listed along with the genus in the Genus column.

OTU	Phylum	Class	Order	Family	Genus	Mean ± SD
*Present on 100% of individuals*						
X926370	Proteobacteria	Gamma	Pseudomonadales	Pseudomonadaceae	*Pseudomonas*	0.003 ± 0.003
X829133	Proteobacteria	Gamma	Aeromonadales	Aeromonadaceae	Unclassified	0.005 ± 0.008
X4453998	Proteobacteria	Beta	Burkholderiales	Comamonadaceae	Unclassified	0.021 ± 0.023
X81358	Proteobacteria	Gamma	Xanthomonadales	Xanthomonadaceae	*Rhodanobacter*	0.003 ± 0.002
X4449458	Proteobacteria	Gamma	Pseudomonadales	Moraxellaceae	*Acinetobacter*	0.009 ± 0.010
X1109251	Proteobacteria	Gamma	Pseudomonadales	Pseudomonadaceae	*Pseudomonas*	0.006 ± 0.011
X410048	Proteobacteria	Gamma	Pseudomonadales	Pseudomonadaceae	*Pseudomonas*	0.007 ± 0.007
X4345285	Firmicutes	Bacilli	Bacillales	Staphylococcaceae	*Staphylococcus*	0.010 ± 0.039
X845178	Proteobacteria	Gamma	Pseudomonadales	Pseudomonadaceae	Unclassified	0.034 ± 0.036
X269930	Proteobacteria	Gamma	Pseudomonadales	Pseudomonadaceae	*Pseudomonas veronii*	0.037 ± 0.022
X394796	Proteobacteria	Gamma	Pseudomonadales	Pseudomonadaceae	*Pseudomonas viridiflava*	0.106 ± 0.112
X4396717	Proteobacteria	Alpha	Rhizobiales	Methylobacteriaceae	*Methylobacterium*	0.004 ± 0.005
X2119418	Proteobacteria	Gamma	Enterobacteriales	Enterobacteriaceae	Unclassified	0.014 ± 0.021
X4419276	Proteobacteria	Gamma	Pseudomonadales	Pseudomonadaceae	Unclassified	0.031 ± 0.055
*Present on 99% of individuals*						
X4323076	Proteobacteria	Beta	Burkholderiales	Oxalobacteraceae	*Janthinobacterium lividum*	0.002 ± 0.002
X1139932	Proteobacteria	Gamma	Xanthomonadales	Xanthomonadaceae	*Stenotrophomonas*	0.012 ± 0.011
X429048	Proteobacteria	Gamma	Xanthomonadales	Xanthomonadaceae	*Stenotrophomonas maltophilia*	0.005 ± 0.005
X4378239	Actinobacteria	Actinobacteria	Actinomycetales	Sanguibacteraceae	*Sanguibacter*	0.072 ± 0.082
X814442	Proteobacteria	Gamma	Enterobacteriales	Enterobacteriaceae	*Citrobacter*	0.009 ± 0.035
X151176	Proteobacteria	Alpha	Rhizobiales	Methylobacteriaceae	*Methylobacterium organophilum*	0.003 ± 0.003
X817734	Proteobacteria	Gamma	Pseudomonadales	Pseudomonadaceae	Unclassified	0.004 ± 0.009
X2468881	Proteobacteria	Gamma	Pseudomonadales	Pseudomonadaceae	*Pseudomonas*	0.004 ± 0.008
X4451011	Proteobacteria	Gamma	Pseudomonadales	Pseudomonadaceae	*Pseudomonas*	0.006 ± 0.005
*Present on 97% of individuals*						
X103411	Proteobacteria	Gamma	Pseudomonadales	Moraxellaceae	*Acinetobacter*	0.003 ± 0.006
X4309301	Firmicutes	Bacilli	Lactobacillales	Streptococcaceae	*Streptococcus*	0.007 ± 0.013
X668514	Proteobacteria	Gamma	Enterobacteriales	Enterobacteriaceae	Unclassified	0.002 ± 0.004
X400315	Proteobacteria	Gamma	Pseudomonadales	Pseudomonadaceae	*Pseudomonas veronii*	0.001 ± 0.001
*Present on 96% of individuals*						
X816702	Proteobacteria	Gamma	Enterobacteriales	Enterobacteriaceae	Unclassified	0.001 ± 0.001
X4456891	Proteobacteria	Gamma	Pseudomonadales	Pseudomonadaceae	*Pseudomonas*	0.001 ± 0.001
X4341734	Actinobacteria	Actinobacteria	Actinomycetales	Microbacteriaceae	Unclassified	0.002 ± 0.002
X1140286	Proteobacteria	Beta	Burkholderiales	Comamonadaceae	*Hylemonella*	0.001 ± 0.002
X102915	Proteobacteria	Alpha	Sphingomonadales	Sphingomonadaceae	*Sphingomonas*	0.004 ± 0.003
X4327233	Actinobacteria	Actinobacteria	Actinomycetales	Microbacteriaceae	Unclassified	0.002 ± 0.003
denovo13279	Bacteroidetes	Sphingobacteriia	Sphingobacteriales	Sphingobacteriaceae	Unclassified	0.014 ± 0.020

Beta diversity varied across sites, elevations, and land use (PERMANOVA all *P* < 0.03; Table 3, Figs. 1D–1F). Elevation explained 7–12% of the variation in bacterial community composition; land use explained less than 5% (all indices; Table 3). This may be in part due to relatively subtle differences in OTU relative abundance between land use types, and stronger differences across elevations. For example, 20 OTUs were identified as differing between land use types based on K–S statistics, but K–S measures associated with these OTUs were all relatively low (range 0.25–0.29; Table 4), indicating that the differences in their empirical distribution functions across land use types were quite small. Most of these K–S OTUs that differentiated the intact and disturbed sites had a higher relative abundance in intact, forested habitats (Fig. 2A). By contrast, for elevational differences, K–S measures for the 18 OTUs identified ranged from 0.47 to 0.58 (Table 5). Most of these OTUs displayed the highest relative abundances at mid-elevations (Fig. 2B).

**Figure 3:**
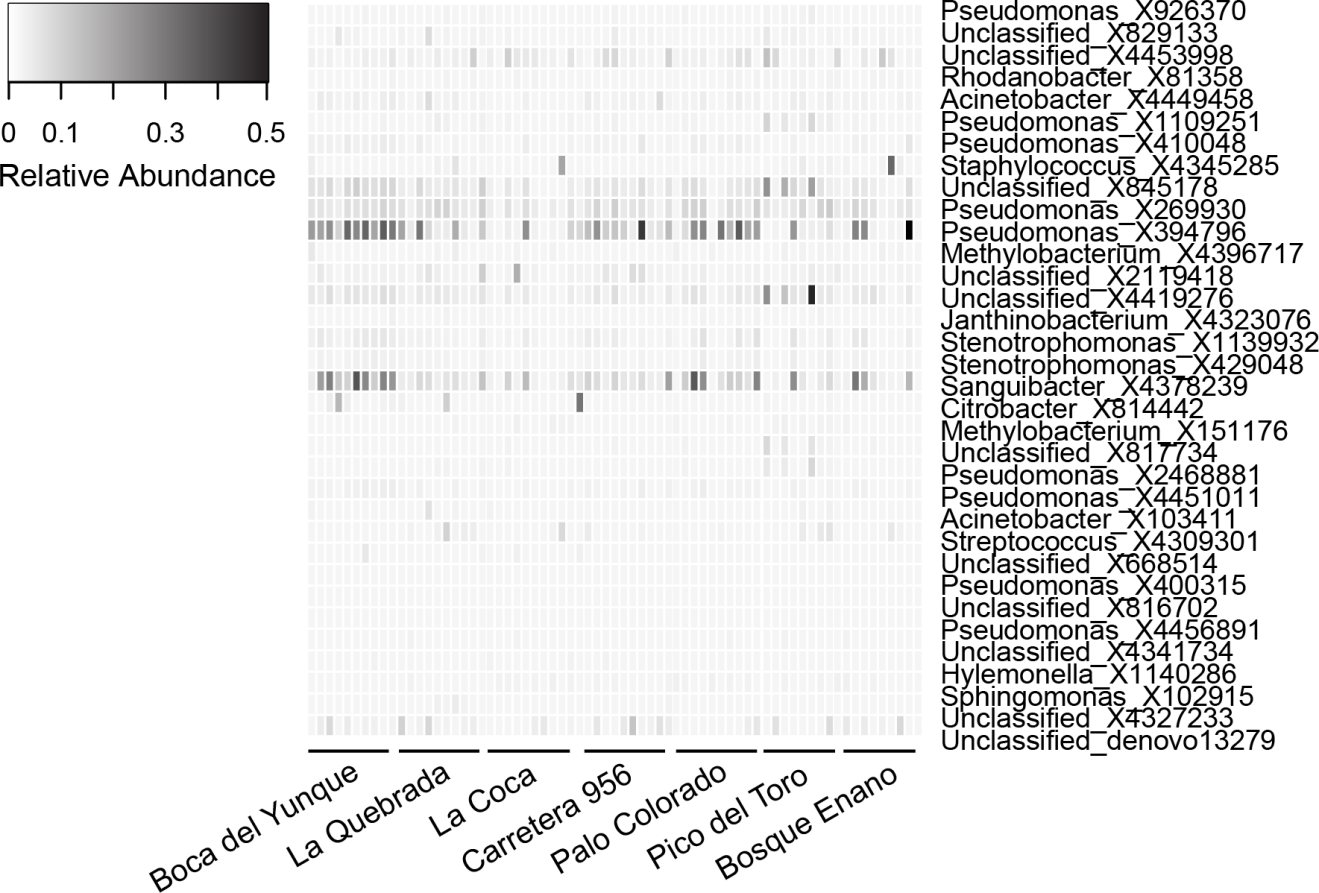
Relative abundance of OTUs (*N* = 34) representing the core microbiota for *Eleutherodactylus coqui*. To be considered part of the core, OTUs had to be present on ≥95% of individuals. OTU relative abundances ranged from 0 to 0.496. Lighter shades indicate lower relative abundances (white relative abundance = 0) and darker shades indicate higher relative abundances (darkest relative abundance = 0.496). Individuals from each site that we sampled are grouped from left to right. See Table 6 for additional details regarding each OTU.

The core microbiome was comprised of 34 OTUs (Table 6). The vast majority (82%) of core OTUs were Proteobacteria; however, at lower taxonomic levels, a variety of bacterial orders (*N* = 11) and families (*N* = 14) were represented. Relative abundances of core OTUs varied considerably across individuals, but were generally low (mean relative abundance of 25/34 core OTUs was ≤0.01%; Table 6, Fig. 3). There were only a few OTUs that displayed higher relative abundances on most individuals, most notably X394796 *Pseudomonas viridiflava* and X4378239 *Sanguibacter sp.* (relative abundance, mean ± sd, X394796: 10.6 ± 11.2% and X4378239: 7.2 ± 8.2%; Fig. 3). No core OTUs were identified by K–S measure as varying across elevation or land use.

## Discussion

Land use change is widely recognized as a major factor impacting biodiversity around the globe. There is increasing evidence linking land use practices to changes in microbiome diversity, especially gut microbiome diversity (Amato et al., 2013; Barelli et al., 2015; Amato et al., 2016; Chang et al., 2016), whereas patterns of microbiome diversity and function along elevation gradients have received less attention (but see Bresciano et al., 2015; Medina et al., 2017; Muletz-Wolz et al., 2017). Our findings reveal subtle, but potentially important, variation in amphibian skin bacterial community structure with site, elevation, and land use.

There are several mechanisms by which these factors could impact skin microbiome diversity. Changes in *E. coqui* bacterial community structure may reflect direct responses of the skin communities to external environmental conditions experienced by the host, or variation in the environmental microbes available to colonize the skin. Studies comparing the diversity of the amphibian skin bacterial communities to that of free-living bacteria in the surrounding environment suggest that many bacteria that reside on amphibian skin can be also be found in the environment, although relative abundances often differ in environmental samples and amphibian skin samples (Kueneman et al., 2014; Walke et al., 2014; Rebollar et al., 2016; Prado-Irwin et al., 2017). This, along with experimental manipulations demonstrating the importance of environmental source pools to the maintenance of skin bacterial diversity (Loudon et al., 2014; Michaels, Antwis & Preziosi, 2014), provide growing evidence for dispersal of bacteria between amphibians and their habitat. Although assessing the environmental microbiota was beyond the scope of this study, it seems reasonable that variation in the diversity of the environmental microbiota contributes to the variation in *E. coqui* skin bacterial communities observed in this study.

Additionally, the differences we observed may be a consequence of altered host physiology or immune function under different environmental conditions. For example, in Neotropical amphibians, elevation (Burrowes, Longo & Rodríguez, 2008; Burrowes et al., 2008; Sapsford, Alford & Schwarzkopf, 2013) and land use (Becker & Zamudio, 2011) are important predictors of the pathogenicity of the chytrid fungus, *Batrachochytrium dendrobatidis*. Bresciano et al. (2015) observed both increased *Bd* infection prevalence and increased presence of *Bd*-inhibiting skin bacteria at high elevations relative to low elevations, linking elevational patterns of pathogen presence in the host with changes in skin bacterial diversity and disease-resistance function. This suggests that there may be stronger selection for more *Bd*-inhibiting bacteria where *Bd* infection prevalence is greater. On the other hand, physiological differences associated with harboring even mild *Bd* infections, as occurs in high (>650 m) but not low elevation populations of *E. coqui* (Burrowes, Joglar & Green, 2004; Burrowes, Longo & Rodríguez, 2008), could manifest as changes to the microbiome. Experimental tests of changes in microbiome diversity point to a role for both direct and host-mediated responses across a wide variety of organisms (Lokmer & Wegner, 2015; Kohl & Yahn, 2016; Zaneveld et al., 2016). Thus, variation in the amphibian skin microbiome likely represents a complex interplay of the effects of both abiotic and biotic conditions on the host and the symbiont community.

One previous study of the microbiome of *E. coqui* (Longo et al., 2015) provides some additional insight into important drivers of bacterial community structure for this species. That study assessed variation in bacterial community diversity across juveniles and adults infected and not infected with *Bd*, and determined that developmental stage was a much more important determinant of alpha and beta diversity than *Bd* infection status (Longo et al., 2015). Based on their findings, Longo et al. (2015) suggested that the environment (what they term “microbial reservoirs”) likely plays a stronger role in shaping the skin microbiota than disease-related factors, such as host immunity. In the present study, no individuals were infected with *Bd*, so we were unable to assess the effects of immediate disease state on microbiome diversity. The absence of *Bd*-infected individuals is most likely due to the timing of the study, which was conducted in the warm-wet season. Previous work suggests that both *Bd* prevalence and infection intensities are lower during this time of the year relative to the cool-dry season (Longo & Burrowes, 2010; Longo et al., 2013; Longo & Zamudio, 2017). However, we expect that *Bd* could be related to microbiome diversity over longer time scales. Historically, *E. coqui* experienced die-offs from *Bd* at higher elevations (Burrowes, Joglar & Green, 2004; Burrowes, Longo & Rodríguez, 2008; Burrowes et al., 2008; Longo et al., 2013), and persisting populations are more vulnerable to the fungus in montane habitats during the dry season (Burrowes et al., 2008; Longo et al., 2013). If these die-off events imposed selection on microbiome diversity, then legacy effects may explain some of the elevational variation in microbiome diversity we observed. Similarly, *Bd* may contribute to among-site variation in the microbiome of the robber frog *Craugastor fitzingeri* from *Bd* endemic versus *Bd* naïve sites in Panama (Rebollar et al., 2016).

Despite the influence of these varied factors on microbial community structure, *E. coqui* appear to maintain a core set of bacterial taxa on their skin. Our characterization of *E. coqui* core taxa is broadly similar to the findings of Longo et al. (2015), who identified five core phylotypes: Actinomycetales, *Stenotrophomonas sp.*, Comamonadaceae, *Staphylococcus sp.*, and Pseudomonadaceae. All of these taxa were represented in our assessment of the core microbiome as well, in addition to many other OTUs. Our understanding of the functional role of the core microbiota of amphibians is limited, but the presence of a core microbiota—which exists in many amphibian species (e.g., Becker et al., 2014; Loudon et al., 2014; Walke et al., 2014)—may imply functional importance. Studies examining the functional role of amphibian skin bacteria have primarily focused on the ability to protect the host against pathogens (e.g., Bletz et al., 2013; Woodhams et al., 2016; Longo & Zamudio, 2017). In this study, many of the core bacteria of *E. coqui* were in genera containing members with known anti-*Bd* properties, including *Acinetobacter*, *Janthinobacterium*, *Pseudomonas*, and *Stenotrophomonas* (Woodhams et al., 2015). However, the skin bacteria may serve other functions. For example, in fish, some skin bacteria may help in the production of the mucosal layer of the skin (Sar & Rosenberg, 1989); this could also be an important function for amphibian skin, especially for terrestrial amphibians in the relatively low humidity conditions typical of disturbed habitat. Additional studies are needed to better understand what role the core and variable components of the microbiota of *E. coqui* may play in allowing them to persist in a variety of habitats and in presence of *Bd*. Moreover, it would be interesting to know if the core microbiota are obtained via different mechanisms than the more variable components. For amphibians like *E. coqui* that provide parental care (Townsend, Stewart & Pough, 1984), there is growing evidence that members of the skin microbiota are shared between parents and eggs (Banning et al., 2008; Walke et al., 2011; Hughey, Delia & Belden, 2017), which could indicate vertical transfer of microbial symbionts.

### Conclusions

Given the functional importance of microbial symbionts, assembly processes are likely to be strongly mediated by the host. This study, however, provides additional evidence of the importance of the environment, even though individual hosts can play a critical role in shaping their bacterial symbiont communities. Future studies should focus on increasing our understanding of the mechanisms by which environmental factors influence the diversity of these communities—via effects on the composition of microbial source pools, alterations to host physiology or immune function, or a combination of these mechanisms—and how this variation ultimately impacts the host organism.

## Additional Information and Declarations

### Field Study Permissions

The following information was supplied relating to field study approvals (i.e., approving body and any reference numbers): This research was approved by the Departamento de Recursos Naturales y Ambientales in Puerto Rico.

### Data Availability

The following information was supplied regarding data availability: Sequence data have been submitted to the NCBI database under accession number SRX2986909.
